# Diagnostic value of skin RT-QuIC in Parkinson’s disease: a two-laboratory study

**DOI:** 10.1038/s41531-021-00242-2

**Published:** 2021-11-15

**Authors:** Anastasia Kuzkina, Connor Bargar, Daniela Schmitt, Jonas Rößle, Wen Wang, Anna-Lena Schubert, Curtis Tatsuoka, Steven A. Gunzler, Wen-Quan Zou, Jens Volkmann, Claudia Sommer, Kathrin Doppler, Shu G. Chen

**Affiliations:** 1 Department of Neurology, University Hospital of Würzburg, Würzburg, Germany; 2 Department of Pathology, Case Western Reserve University School of Medicine, Cleveland, OH USA; 3 Department of Neurology, Case Western Reserve University School of Medicine, Cleveland, OH USA; 4 Department of Neurology, University Hospitals Cleveland Medical Center, Cleveland, OH USA

**Keywords:** Diagnostic markers, Parkinson's disease, Diagnostic markers

## Abstract

Skin α-synuclein deposition is considered a potential biomarker for Parkinson’s disease (PD). Real-time quaking-induced conversion (RT-QuIC) is a novel, ultrasensitive, and efficient seeding assay that enables the detection of minute amounts of α-synuclein aggregates. We aimed to determine the diagnostic accuracy, reliability, and reproducibility of α-synuclein RT-QuIC assay of skin biopsy for diagnosing PD and to explore its correlation with clinical markers of PD in a two-center inter-laboratory comparison study. Patients with clinically diagnosed PD (*n* = 34), as well as control subjects (*n* = 30), underwent skin punch biopsy at multiple sites (neck, lower back, thigh, and lower leg). The skin biopsy samples (198 in total) were divided in half to be analyzed by RT-QuIC assay in two independent laboratories. The α-synuclein RT-QuIC assay of multiple skin biopsies supported the clinical diagnosis of PD with a diagnostic accuracy of 88.9% and showed a high degree of inter-rater agreement between the two laboratories (92.2%). Higher α-synuclein seeding activity in RT-QuIC was shown in patients with longer disease duration and more advanced disease stage and correlated with the presence of REM sleep behavior disorder, cognitive impairment, and constipation. The α-synuclein RT-QuIC assay of minimally invasive skin punch biopsy is a reliable and reproducible biomarker for Parkinson’s disease. Moreover, α-synuclein RT-QuIC seeding activity in the skin may serve as a potential indicator of progression as it correlates with the disease stage and certain non-motor symptoms.

## Introduction

Clinical diagnosis of idiopathic Parkinson’s disease (PD) is primarily based on the presence of a parkinsonian syndrome along with supportive signs and absence of red flags suggestive of an alternate diagnosis^1^. The wide range of clinical signs and their evolution during the disease course complicate the early and differential diagnosis. Retrospective neuropathological studies have found a highly variable accuracy of clinical diagnosis between 46 and 95%^2^. As understanding of the disease evolves, additional objective diagnostic markers, such as nuclear brain imaging, have been incorporated into the diagnostic criteria. However, due to the lack of standardization of feasible and easily reproducible assays, tissue biomarkers have not yet been included.

Aggregation of α-synuclein (α-syn) is considered the histoneuropathological hallmark of PD. In the past decade, depositions of α-syn have been observed premortally in peripheral nerves in a variety of tissues, including skin that is easily accessible^3–5^. Several studies reported immunohistochemical (IHC) detection of phosphorylated α-syn in dermal nerve fibers^5–8^. However, in spite of numerous follow-up studies that included different protocols for detection of α-syn aggregates and oligomers by IHC and proximity ligation assay, the method has not entered clinical practice^7–13^. Immunohistochemical detection of dermal α-syn is time-consuming, quantification is difficult, and the extent of deposits does not correlate with disease progression^5,6^. A more practical and validated method that allows for rapid analysis of a large number of samples is needed. Ideally, quantification of this biomarker should correlate with disease progression.

Real-time quaking-induced conversion (RT-QuIC) assay, based on prion-like seeding activity of α-syn, has already shown promise in detecting α-syn aggregates in the brain and cerebrospinal fluid (CSF) of patients with synucleinopathies with high accuracy^14,15^. A recent study with skin-based RT-QuIC and protein misfolding cyclic amplification assays provided the proof-of-concept evidence that skin α-syn aggregation seeding activity could be a novel biomarker for PD, but data on the validation of the method and its correlation with clinical measures of PD are lacking^16^.

We therefore conducted a structured two-center study to assess the inter-laboratory reliability and reproducibility of the skin RT-QuIC assay for the diagnosis of PD and to examine its correlation with symptoms and disease progression in order to explore its potential use as a peripheral tissue biomarker.

## Results

### Patients and controls

Thirty-four patients with PD and 30 controls were included in this prospective study. A total of 117 individual biopsies from 34 PD patients as well as 81 biopsies from 30 control subjects were collected. Age and gender did not differ between the PD and control groups. Demographic and clinical data are summarized in Table 1.

**Table 1:** Demographic and clinical data of patients and controls.

Characteristic	PD (*n* = 34)	Controls (*n* = 30)	*p* value
Age (years), mean ± SD	67.1 ± 8.6	63.5 ± 7.9	0.08
Male:female, *n*	24:10	17:13	0.53
Disease duration (years), mean ± SD	11.7 ± 6.9	NA	NA
Age at diagnosis (years), mean ± SD	55.4 ± 12	NA	NA
Hoehn und Yahr stage, mean ± SD	2.5 ± 0.8	NA	NA
NMSS score sum, mean ± SD	46.26 ± 33	NA	NA
MCI, *n* (%)	11 (33%)	NA	NA
DBS surgery, *n* (%)	17 (50%)	NA	NA
RBD, positive screening question, *n* (%)	20 (59%)	2 (6.7%)	<0.001

*SD* standard deviation, *PD* Parkinson’s disease, *NMSS* Non-Motor Symptom Scale, *MCI* mild cognitive impairment, *DBS* deep brain stimulation, *RBD* REM sleep behavior disorder, *NA* not applicable.

### α-Syn seeding activity detected by RT-QuIC is increased in skin biopsies from PD patients

**Fig. 1:**
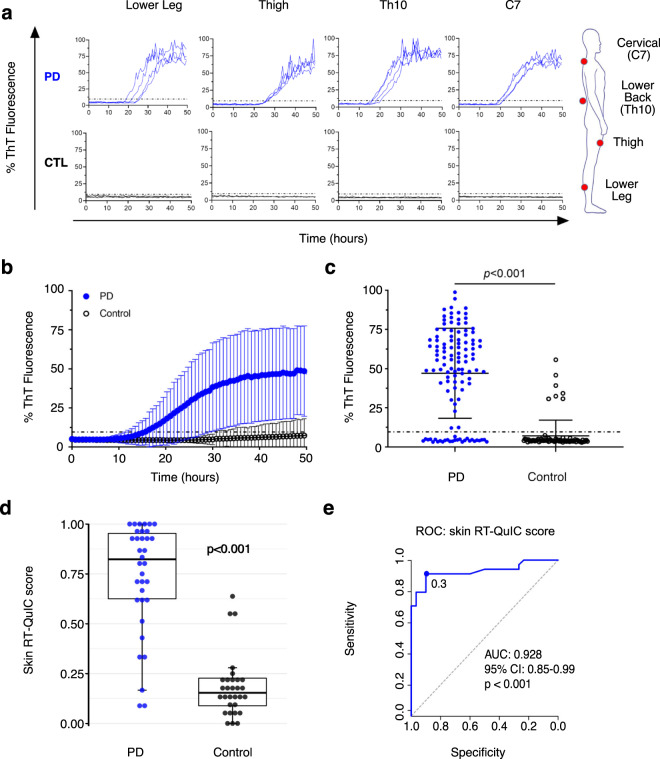
RT-QuIC detection of α-syn seeding activity in skin biopsies from PD patients and control subjects. **a** α-Syn RT-QuIC assay of multiple skin biopsies from a PD patient (PD10) and a control subject (CTL2). Four skin biopsy sites at the level of C7 (neck), Th10 (lower back), thigh, and lower leg are indicated. Shown are individual ThT fluorescence responses over time for each skin sample tested in quadruplicate. The dotted line indicates threshold at 9.6%. A marked increase of ThT fluorescence over time is found in patient samples but not in the control samples. **b** Cumulative results of blinded testing of all skin biopsies by α-syn RT-QuIC (Cleveland center). Mean values (±SD) of percentage of ThT fluorescence of all biopsies tested in quadruplicate are clearly increased in PD patients (*n* = 34) compared to control subjects (*n* = 30). The dotted line indicates threshold at 9.6%. **c** Scatter graph of final percentage of ThT fluorescence intensities (50 h) for all individual biopsies. Average ThT fluorescence intensities of skin biopsies from PD patients (*n* = 34 with 117 biopsies) are significantly higher (*p* < 0.001) than those from control subjects (*n* = 30 with 81 biopsies). Dots represent values of individual biopsies, the dotted line indicates threshold at 9.6%. Group mean ± SD are represented by solid lines. **d** Skin RT-QuIC scores in PD patients (*n* = 34) and control subjects (*n* = 30). Scores are pooled from both Cleveland and Würzburg centers. A box plot demonstrates significantly higher scores in PD compared to controls (*p* < 0.001). The horizontal line in the box plot marks the median, the box represents the interquartile range (IQR), and the whiskers extend to the most extreme data point, which is no more than 1.5 times of IQR. **e** Receiver operating characteristic (ROC) curve and the corresponding area under the curve (AUC) for skin RT-QuIC scores. ROC analysis demonstrates an optimal RT-QuIC score threshold of 0.3 that would result in a 91.2% sensitivity and 90.0% specificity. CI confidence interval.

We performed RT-QuIC assay of multiple skin biopsies per subject, from subjects with PD and controls, in a blinded prospective study. As exemplified in Fig. 1a, RT-QuIC assay readily detected α-syn seeding activity as shown by the enhanced ThT fluorescence in all four skin biopsies collected from a PD patient but not in those from a control subject. Overall, RT-QuIC analyses of the whole cohort confirmed a progressive increase of ThT fluorescence of skin biopsies over time in the PD group (*n* = 34), crossing the cut-off threshold at a mean of 18 h (±5) and reaching 50% of the maximum fluorescence at 26 h (±5), while ThT fluorescence of skin biopsies of the control group (*n* = 30) remained much lower (Fig. 1b, Cleveland data). Accordingly, the final ThT fluorescence intensities (50 h) were significantly higher in the PD group (47.2 ± 24.5%) than in the control group (7.92 ± 9.87%), *p* < 0.001, Fig. 1c). The calculated skin RT-QuIC scores of combined data from multiple skin sites of each participant were markedly higher in the PD group compared to the control group (*p* < 0.001, Fig. 1d and Table 2), reflecting significantly higher overall skin α-syn seeding activity in PD.

**Table 2:** Results of α-syn RT-QuIC of skin biopsies from both centers.

	PD	Control	*p* value	*N*	Cohen’s *d* [95% CI]	
*Final percentage of ThT fluorescence of biopsies, mean (SD)*						
Cleveland						
C7	46.1 (28.2)	6.52 (9.2)	<0.001	64	1.84 [1.25, 2.42]	
Th10	43.3 (30.3)	5.39 (6.3)	<0.001	59	1.64 [1.04, 2.23]	
Thigh	46.1 (30.2)	10.6 (15.2)	<0.001	57	1.42 [0.82, 2.00]	
Lower leg	55.3 (25.9)	5.34 (NA)	NA	18	NA	
Total (average across biopsy sites)	47.2 (24.5)	7.92 (9.9)	<0.001	64	2.05 [1.44, 2.66]	
Würzburg						
C7	29.3 (23.9)	3.02 (5.8)	<0.001	64	1.47 [0.91, 2.02]	
Th10	35.3 (29.1)	2.54 (2.9)	<0.001	59	1.50 [0.91, 2.07]	
Thigh	33.3 (24.3)	3.42 (4.5)	<0.001	57	1.59 [0.98, 2.19]	
Lower leg	31.1 (20.1)	1.38 (NA)	NA	17	NA	
Total (average across biopsy sites)	32.6 (21.3)	3.12 (3.8)	<0.001	64	1.86 [1.27, 2.45]	

RT-QuIC scores, median [IQR]	PD	Control	*p* value	*N*	Cohen’s *d* [95% CI]	
Combined results of both sites						
Skin RT-QuIC score	0.82 [0.62; 0.95]	0.15 [0.09; 0.23]	<0.001	64	2.45 [1.79, 3.10]	
Neck C7 RT-QuIC score	0.94 [0.58; 1]	0.12 [0.00; 0.12]	<0.001	64	2.06 [1.44, 2.66]	
Lower back Th10 RT-QuIC score	0.88 [0.25; 1]	0.12 [0.12; 0.25]	<0.001	59	1.66 [1.05, 2.25]	
Thigh RT-QuIC score	0.88 [0.62; 1]	0.19 [0.12; 0.38]	<0.001	58	1.81 [1.19, 2.42]	

RT-QuIC results of participants, *n* (%)	PD		Control			
Negative (skin RT-QuIC score ≤0.25)	3 (8.8%)		26 (86.7%)			
Intermediate (0.25 < skin RT-QuIC score < 0.5)	3 (8.8%)		1 (3.3%)			
Positive (skin RT-QuIC score ≥0.5)	28 (82.4%)		3 (10.0%)			

Outcome	Degree of agreement		*p* value			*N*
Inter-laboratory agreement						
Patients: positive/negative	*κ* = 0.861		<0.001			64
Biopsy: positive/negative	*κ* = 0.783		<0.001			198
RT-QuIC scores of biopsies (0–1)	*κ*_weighted_ = 0.881		<0.001			198
	Spearman’s *ρ* = 0.82, 95% CI [0.75, 0.87]		<0.001			

Cohen’s *d* reflect effect sizes and were calculated as the mean difference between PD and control groups divided by the pooled standard deviation.

*N* total number of subjects or biopsies for each comparison, *SD* standard deviation, *PD* Parkinson’s disease, *NA* not applicable, *IQR* interquartile range, *κ* Cohen’s kappa coefficient.

Based on the cut-off of the skin RT-QuIC, 28 PD patients (82.4%) were classified as positive, 3 (8.8%) as intermediate (Fig. 1d). In the control group, only 3 patients (10%) were classified as positive, 1 as intermediate (3.3%). This translated to an 88.9% diagnostic accuracy (sensitivity 90.9%, specificity 86.7%) if intermediate results were considered equal to positive. When intermediate results were considered as negative, the resulting diagnostic accuracy was lowered to 85.7% with decreased sensitivity (81.8%), but slightly higher specificity (90%). A post hoc receiver operating characteristic analysis revealed an optimal skin RT-QuIC score cut-off at 0.3 showing a maximum 90.0% specificity and 91.2% sensitivity (Fig. 1e), with 31/34 positive PD patients, and 27/30 controls remaining negative. Combined results of two sites (C7 and thigh) reached 85% sensitivity, while showing an even superior (93%) specificity (as omitting a supposedly false-positive Th10 site changed the final result of subject CTL30 to negative).

**Fig. 2:**
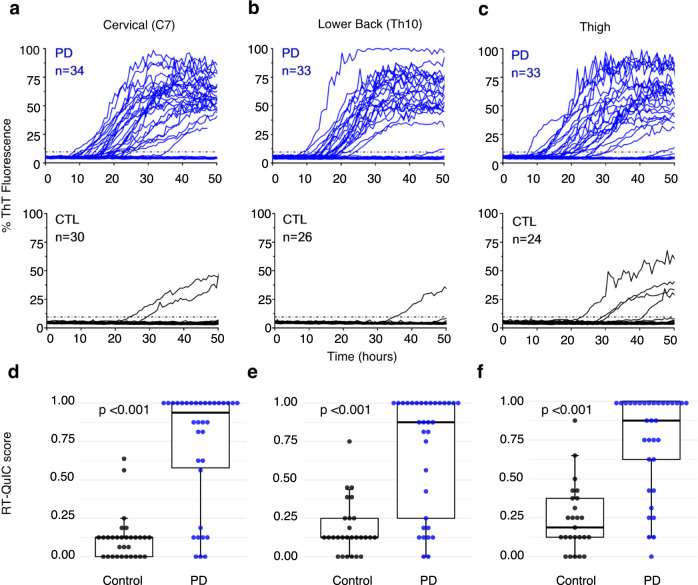
Higher α-syn seeding activity in skin biopsies from different anatomical sites in PD patients as compared to control subjects. **a**–**c** show RT-QuIC reactivity in skin biopsies of PD patients and controls collected at the locations of cervical C7, lower back Th10, and thigh, respectively. ThT fluorescence from individual skin biopsies is more intense and occurs more frequently in PD (blue curves) than in controls (CTL, black curves) above the threshold of 9.6% (dotted lines). Raw data are taken from the Cleveland center. **d**–**f** illustrate the RT-QuIC scores for individual skin biopsies in PD and control groups collected at cervical C7 (**d**), thoracal Th10 (**e**), and thigh (**f**). Scores are pooled from both Cleveland and Würzburg centers. The box plots demonstrate significantly higher scores in PD patients compared to controls (*p* < 0.001). The boxes represent the interquartile range (IQR) with the median line and the whiskers extending to the most extreme data point, which is no more than 1.5 times of IQR.

**Fig. 3:**
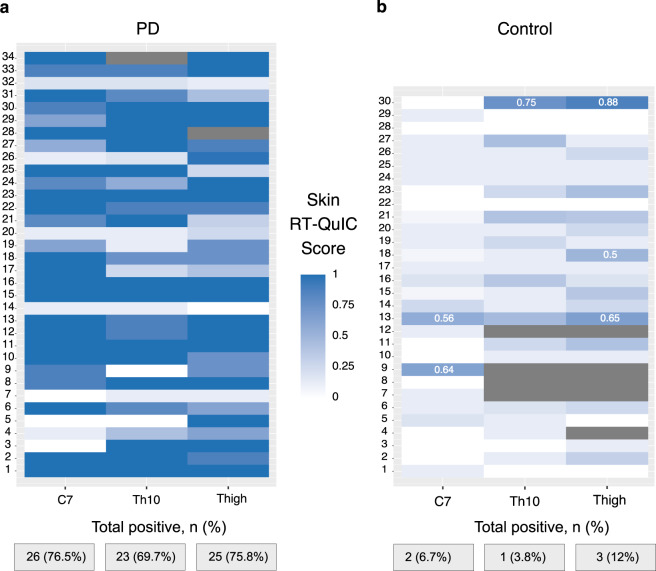
α-syn seeding activity is conserved across multiple biopsy sites in PD and is universally low in controls. Distribution of skin RT-QuIC scores ranging from 0 (white) to 1 (blue) across cervical (C7), thoracal (Th10), and thigh sites in PD patients (**a**) and control subjects (**b**). The graphs show a mostly uniform presence of skin α-syn seeding activity in individual biopsy sites in PD patients, and only sparsely in single biopsy sites in controls. Subjects are listed vertically. The total number (%) of biopsies with positive RT-QuIC scores (≥0.5) at each site for the PD and control groups is indicated below each graph. Missing biopsy spots are shaded gray. Scores are pooled from both Cleveland and Würzburg centers.

ThT fluorescence induced by skin biopsies from PD patients was higher and occurred more frequently across all anatomical sites (C7, Th10, thigh) as compared to the control subjects (*p* < 0.001, Table 2 and Fig. 2a–c). Supporting extensive RT-QuIC positivity in PD, skin RT-QuIC scores were significantly higher in the PD group than in the control group in all sites (Fig. 2d–f and Table 2, *p* < 0.001), with the best separation between the PD and control group in C7 (Fig. 2d). In positive PD patients (*n* = 28), RT-QuIC positivity was generally conserved across multiple biopsy sites (Fig. 3), 19 patients (68%) were positive at all sites, while all 28 patients (100%) had at least two positive sites. In contrast, RT-QuIC positivity was only sparsely observed in 3/30 control subjects (10%) at individual sites.

### High inter-laboratory reproducibility of RT-QuIC

Inter-laboratory agreement of RT-QuIC results (positive/negative) was reached in 92.2% of cases and in 88.9% of biopsies (see Table 2). To explore the source of discrepancies, such as due to sample differences (different amount of α-syn seeds in the halves of the biopsy specimen or differences in homogenization procedure) or differences in the RT-QuIC assay protocols, a subgroup of lysates was exchanged (*n* = 126 (63.6%) of 198 total). Same-source lysates analyzed at both laboratories confirmed an excellent agreement between the assays (*κ* = 0.84, *p* < 0.001). The comparison of RT-QuIC of skin lysates originating from different halves of the same biopsy showed a substantial reproducibility in both assays (*κ*_weighted_ = 0.7, *p* < 0.001).

### Clinical characteristics in patients with low α-syn seeding activity and in controls with positive RT-QuIC scores

PD patients with negative (*n* = 3) and intermediate (*n* = 3) RT-QuIC results were re-evaluated, but no red flags indicating an alternative diagnosis were found. All but one of the patients fulfilled the International Parkinson and Movement Disorder Society (MDS) criteria for a clinically established PD. Notably all 6 patients had a normal Montreal cognitive assessment (MoCA) and a significantly lower burden of non-motor symptoms (NMSs) (Non-Motor Symptoms Scale (NMSS) scores of 31.5 (9.38) compared to 49.4 (35.5) in positive patients, *p* = 0.027). In the RT-QuIC-negative patient with probable PD, only one supportive criterion (response to levodopa) could be identified, while rest tremor, dyskinesia, and olfactory loss (verified by normal Sniffin’ sticks assessment) were lacking, and disease duration was just 1 year. In one of the intermediate cases, a bloody lysate appearance was documented, possibly leading to a false-negative result^17^.

Among the three control subjects with a positive RT-QuIC score, one (CTL30; Fig. 3 and Supplementary Table 1) had olfactory dysfunction (anosmia on Sniffin’ sticks assessment) and possible rapid eye movement sleep behavior disorder (RBD) as reported in the single-question screening. The second control subject (CTL13) reported RBD. The third control person (CTL9) reported no signs suggesting prodromal PD apart from urinary dysfunction, depression, and idiopathic small fiber neuropathy. Notably, in the latter case only the lysate from one of the halves of the biopsy tested positive in both Würzburg and Cleveland laboratories, whereas the other half remained negative.

### α-Syn seeding activity in skin biopsies correlates with disease duration and stage

**Fig. 4:**
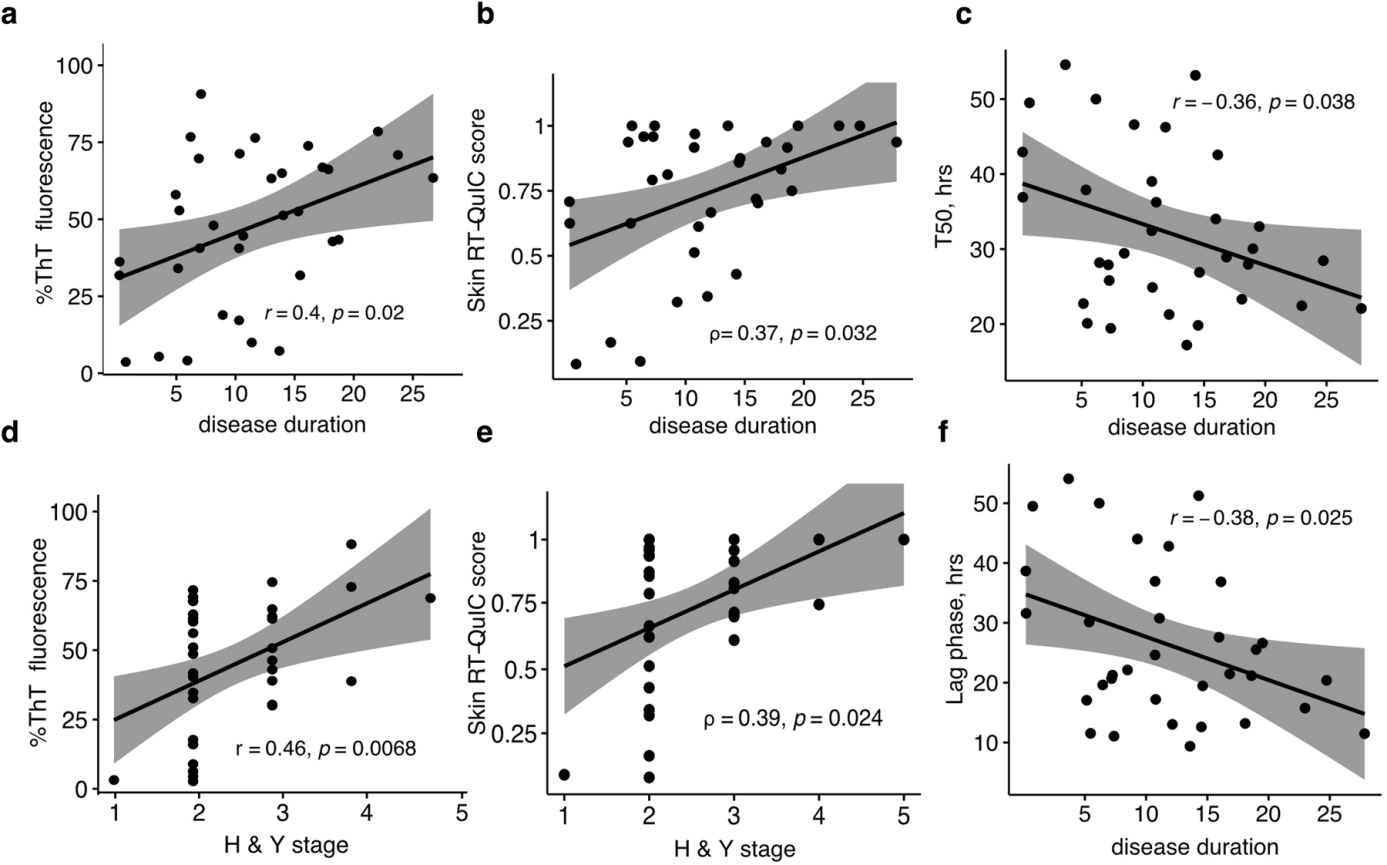
Skin RT-QuIC results correlate with disease progression. Raw RT-QuIC assay parameters correlate with PD duration: longer disease duration is associated with a higher final percentage of ThT fluorescence (**a**), shorter duration of T50, time needed to reach 50% aggregation (**c**), and shorter lag phase (**f**). **d** Higher H&Y stage is associated with a higher final percentage of ThT fluorescence. Raw data (**a**, **c**, **d**, **f**) are taken from the Cleveland assay. **b**, **e** Skin RT-QuIC scores (0–1) increase with longer disease duration and higher H&Y stage. Linear regression lines with 95% confidence interval (gray shade) are shown. Scores are pooled from both Cleveland and Würzburg centers.

Disease duration positively correlated with the final percentage of ThT fluorescence (*r* = 0.4, *p* = 0.02) and negatively correlated with T50 (time to reach half maximum fluorescence, *r* = −0.36, *p* = 0.038) and lag phase (time to cross threshold fluorescence, *r* = −0.38, *p* = 0.025), as demonstrated in Fig. 4a, c, f (data from Cleveland center shown). Moreover, Hoehn and Yahr (H&Y) stage moderately correlated with the final percentage of ThT fluorescence (*r* = 0.46, *p* = 0.0068, Fig. 4d, Cleveland center). Finally, the skin RT-QuIC score as an integrated measure of α-syn seeding activity of both Cleveland and Würzburg centers also positively correlated with both the disease duration (*ρ* = 0.37, *p* = 0.032) and the H&Y stage (*ρ* = 0.39, *p* = 0.024, Fig. 4b, e).

**Fig. 5:**
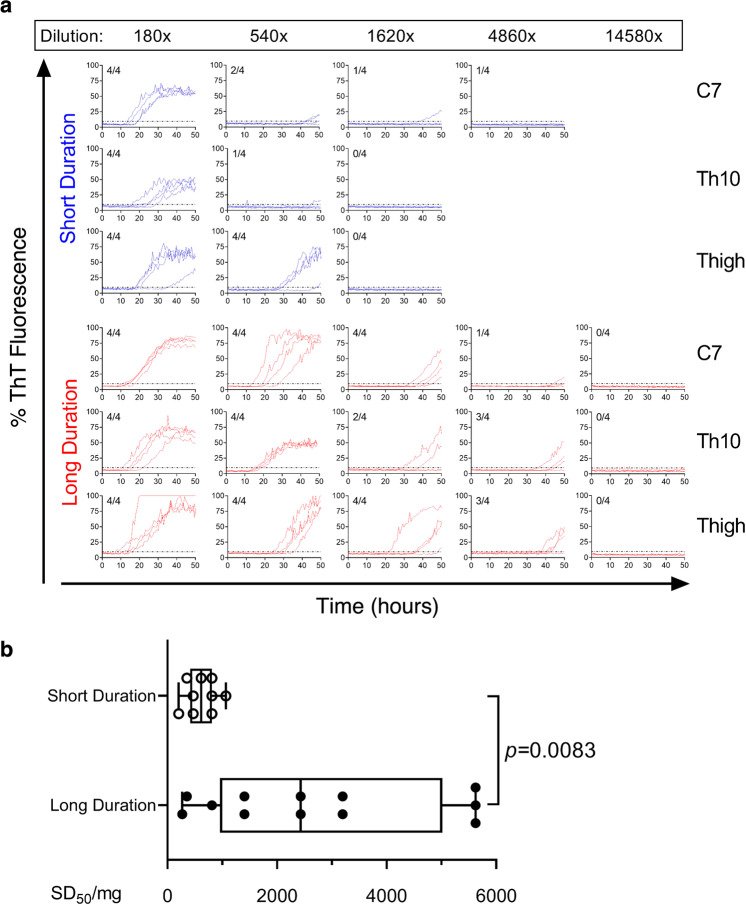
Comparisons of α-syn seeding activity in skin biopsies of PD patients with different disease duration through endpoint dilution analyses. **a** RT-QuIC spectra of serially endpoint diluted skin biopsies collected at C7, Th10, and thigh from a PD patient with a short (7 years, blue curves) or long (28 years, red curves) disease duration. Dilution folds are indicated on the top. Shown are ThT fluorescence traces from four replicate reactions at the designated dilutions. The fractions in the upper left corner of each graph indicate the positive/total replicate reactions. Dotted lines indicate a threshold of 9.6%. **b** Values of SD_50_/mg obtained from Spearman–Kärber endpoint analyses for skin biopsies from all anatomic sites of PD patents with short (5–7 years, *n* = 3) or long (15–28 years, *n* = 4) disease duration. A box plot demonstrates significantly higher SD_50_/mg in PD patients with long disease duration compared to those with short duration (*p* = 0.0083, Mann–Whitney *U* test). The boxes represent the interquartile range with the median line and the whiskers extending to the most extreme data points.

To further evaluate whether a longer disease duration was associated with a higher burden of peripheral α-syn aggregates, Spearman–Kärber analysis of endpoint dilutions was utilized to determine the seeding dose (SD_50_) at which 50% of replicate reactions were positive. This allowed for a quantitative comparison of α-syn seeding activity (in SD_50_ units) among specimens from different patients, similar to the measures used in bioassays^18^. Serial dilutions of skin biopsies at C7, Th10, and thigh from PD patients with shorter (5–7 years, *n* = 3) and longer (15–28 years, *n* = 4) disease duration were performed. Parallel comparisons revealed that α-syn seeding activity persisted in more diluted skin lysates in patients with a long disease duration as compared to those with a short duration (Fig. 5a), with SD_50_ values being fourfold higher in the long duration patients (Fig. 5b; median SD_50_/mg, 2430 units for long duration vs 616 units for short duration; *p* = 0.0083).

### Higher α-syn seeding activity is found in patients with certain NMSs

**Fig. 6:**
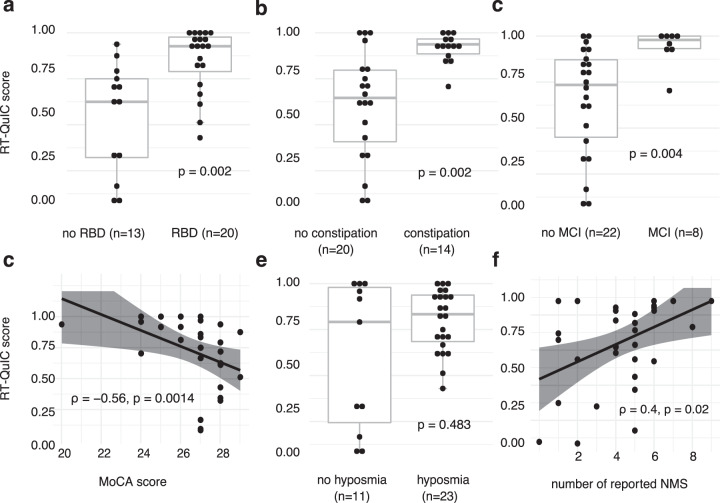
Skin RT-QuIC scores correlate with non-motor symptom burden. **a**–**c**, **e** Box plots illustrating differences in skin RT-QuIC scores between subgroups reporting certain non-motor symptoms. Skin RT-QuIC scores (vertical axis: 0–1) pooled from both centers are higher in patients with possible RBD (**a**), constipation (**b**), and MCI (**c**). The skin RT-QuIC scores are not significantly higher in patients reporting hyposmia (**e**). The boxes represent the interquartile range (IQR), the middle line of each box indicates the median value with the median line and the whiskers extending to the most extreme data point, which is no more than 1.5 times of IQR. Values of individual patients are illustrated by dots. **d**, **f** Scatter plots illustrating correlations of skin RT-QuIC scores with non-motor symptoms. The skin RT-QuIC score negatively correlated with MoCA scores (**d**) and positively with the total number of reported key non-motor symptoms (**f**), respectively. A linear model regression line is shown, the gray shade represents the 95% confidence interval.

The skin RT-QuIC score was higher in PD patients who reported RBD (0.93 [0.8; 0.98] vs 0.63 [0.32; 0.75], *p* < 0.01), constipation (0.94 [0.89; 0.97] vs 0.65 [0.41; 0.8], *p* < 0.01), and in patients with mild cognitive impairment (MCI) (0.98 [0.93; 1] vs 0.73 [0.45; 0.87]), *p* < 0.01) (Fig. 6a–c). The MoCA score negatively correlated with the skin RT-QuIC score (*ρ* = −0.56, *p* = 0.0014, Fig. 6d). Skin RT-QuIC score was not significantly higher in patients who reported hyposmia on NMSS (Fig. 6e). While the correlation between the skin RT-QuIC score and total NMSS score was not significant, the number of reported key NMSs (orthostatic dysfunction, restless legs syndrome, constipation, urinary dysfunction, hyposmia, RBD, insomnia, day-time sleepiness, depression, and MCI) positively correlated with the skin RT-QuIC score (*ρ* = 0.4, *p* = 0.02, Fig. 6f). We did not find a significant difference in skin RT-QuIC score between motor subtypes of PD (the tremor-dominant PD (0.78 [0.62; 0.86] vs the akinetic-rigid PD (0.86 [0.67; 0.97], *p* = 0.705).

## Discussion

We conducted a dual-center comparison study of a skin RT-QuIC assay, demonstrating excellent diagnostic accuracy and inter-laboratory reproducibility for detection of skin α-syn seeding activity in PD. RT-QuIC results also correlated with progression markers and NMSs of PD, suggesting that RT-QuIC has potential value as a biomarker in PD.

The diagnostic accuracy in our study was up to 88.9% with sensitivity of 81.8–90.9% and specificity of 86.7–90% (depending upon whether intermediate results were counted as positive or negative). The overall higher sensitivity compared to prior IHC studies likely indicates that RT-QuIC can detect IHC undetectable α-syn aggregates^5,9,19^. A side-to-side comparison with IHC could provide a more reliable comparison of the methods as well as some insight into the type of affected fibers, which is known to differ between synucleinopathy subtypes^20–22^ and should be addressed in future studies. Moreover, RT-QuIC assay results were highly reproducible in our inter-laboratory comparison compared to IHC, where sensitivity of phosphorylated α-syn detection varied widely between groups^6,23^. Some minor discrepancies in the results were present, possibly due to slight differences in the RT-QuIC assay protocols.

Skin α-syn seeding activity was nearly ubiquitously present in PD. The most proximal (C7) site showed a slightly superior accuracy, similar to prior studies in IHC^5,6^. However, likely due to an overall higher sensitivity of RT-QuIC, we did not find a striking difference in positivity of proximal to distal sites. The lower back site (Th10) was slightly less sensitive, probably due to higher thickness of derma that complicates the separation of the biopsy punch ultimately leading to a smaller biopsy size. Sensitivity of each of the sites in isolation was still substantially inferior to the evaluation of all sites combined. The need to perform multiple punches to reach high diagnostic certainty is therefore a limitation of this approach. However, retrospectively, combined results of the two sites that showed highest accuracy (C7 and thigh) still allowed to reach 85% sensitivity and 93% specificity. The diagnostic accuracy from this relatively small trial should be validated in a larger cohort.

The skin α-syn RT-QuIC seeding activity moderately correlated with H&Y stage and duration of disease. However, the correlation is limited by low number of patients in advanced disease stages and might have biased the lower diagnostic certainty in patients with short disease duration. A correlation of skin α-syn pathology with the H&Y stage was previously reported in a subset of IHC trials using highly diluted primary antibodies against total α-syn^10,11^, possibly causing a preferential binding to the aggregated over the physiological α-syn^9,24^. IHC studies that used antibodies against the phosphorylated form of α-syn could not reproduce this finding^5,6^. However, detection of dermal α-syn aggregates by IHC may be biased by the patchy distribution of α-syn aggregates in the skin resulting in sampling errors, as only a limited number of skin sections can be analyzed. In contrast, RT-QuIC allows for the analysis of whole-biopsy lysates. One of the α-syn seeding studies of CSF^25^ found a correlation with H&Y stage, while others could not confirm it^26,27^. Interestingly, our serial endpoint dilution analyses revealed that skin biopsies in PD patients with longer disease duration had higher RT-QuIC seeding units than those with shorter duration, suggesting enhanced accumulation of aggregated α-syn in peripheral tissues during protracted disease course and potential use of skin RT-QuIC to monitor progression. The correlations with disease progression were moderate and ultimately prospective studies with biopsy assessments at multiple time points would be needed to further validate a correlation with the disease stage and duration.

Clinical relevance of skin RT-QuIC is further supported by the correlation of the skin RT-QuIC score with NMSs. Dermal α-syn deposition in PD is known to primarily affect the autonomic nervous system^5,20,22^ and was previously shown to be associated with dysautonomia^10^. In a recent study, peripheral α-syn deposition assessed in the gastrointestinal system and skin both correlated with Lewy body stage in the brain^28^. Therefore, involvement of different parts of the peripheral nervous system possibly occurs in parallel, and this could explain correlation of constipation with skin α-syn aggregation in our study. RBD is associated with early dermal α-syn deposition detectable by IHC, as it has been found in more than half of prodromal RBD patients^19,29^. In one study, dermal α-syn deposition in RBD was even higher than in PD^30^, suggesting a subgroup with a distinct disease evolution. Supporting this, PD patients suffering from RBD have been reported to have more α-syn lesions in the brain^31^ and higher α-syn RT-QuIC seeding activity in CSF^27^. RBD and constipation are the most common prodromal symptoms of PD^32^ and are both associated with a faster progression and presence of MCI^33^. A higher baseline α-syn seeding activity in CSF was recently shown to be predictive of cognitive decline^34^. Similarly, all three non-motor features of RBD, cognitive impairment, and dysautonomia have been shown to be the key determinants of clustering into a “diffuse malignant” phenotype with higher symptom burden and faster disease progression^35^. Correlation of peripheral, in this case dermal, α-syn pathology with “central” symptoms (RBD and MCI) further supports the systemic nature of α-syn deposition in both central and peripheral tissues as suggested by pathological studies^8,28^. Higher skin α-syn seeding activity as an indicator of higher peripheral, and possibly overall load of α-syn aggregates, in patients with a “malignant phenotype” may imply a potential use of our skin RT-QuIC assay to identify subgroups of PD with different rates of disease progression.

While RT-QuIC supported the clinical diagnosis in most cases, a subgroup of PD patients showed no or low α-syn seeding activity. Only in one of these cases, blood contamination was apparent in the lysates possibly inhibiting aggregation^17^. Moreover, five of these patients reached the highest degree of diagnostic certainty according to MDS criteria, making a false clinical diagnosis unlikely. RT-QuIC-negative PD patients may represent a suggested central nervous system-first phenotype^36^, in which the basal ganglia pathology is postulated to appear prior to the involvement of the peripheral nervous system. This is supported by a lower occurrence of NMSs in these patients. Alternatively, these could be patients in whom α-syn pathology is not consistently found, as in some genetic forms with Parkin, PINK1, and LRRK2 mutations^37,38^. Though none of the patients were genetically tested, 2 of the RT-QuIC-negative patients had an early-onset disease (at 37 and 42 years), and 1 had a positive family history. Moreover, four out of six patients with negative or intermediate RT-QuIC results had prior deep brain stimulation (DBS) surgery, and patients with genetic parkinsonism were previously shown to be overrepresented in the DBS collective due to the predominant motor levodopa-responsive symptoms^39^. Similar to our observations, in a recent CSF RT-QuIC study, three PD patients who tested negative exhibited a remarkably mild disease severity^40^. The absence of α-syn seeding activity in the skin may thus suggest lack of involvement of the peripheral nervous system or absence of α-syn pathology altogether in a small subgroup of PD patients.

Two control subjects reported possible RBD on screening, and both were positive in RT-QuIC, indicating a possible prodromal synucleinopathy. RBD is the most reliable prodromal PD manifestation and is associated with α-syn deposition in dermal autonomic nerves^19,29^. In an earlier CSF study^25^, two of the four control subjects with positive RT-QuIC were found to develop PD several years after CSF sampling, supporting the ability of the α-syn seeding assay to detect prodromal PD stages more often than older methods, such as IHC. This could have great value for enrollment of prodromal PD subjects in neuroprotective trials. It should be noted that skin RT-QuIC scores of control subjects with positive RT-QuIC were still markedly lower than the average score of the PD group.

An important limitation of our study that must be addressed in the future is omission of patients with atypical parkinsonism. Skin RT-QuIC appears particularly interesting for differential diagnosis with multiple system atrophy, where α-syn is known to preferentially deposit in somatosensory fibers in contrast to autonomic fibers in PD^20–22^ and was recently shown to be biochemically distinct in a CSF study^41^.

In summary, our study shows that the skin RT-QuIC assay achieves a high diagnostic accuracy for PD, similar to the more intensively studied CSF^14,25,26^. Skin biopsy has the advantage of minimal invasiveness that allows for repeated sampling in longitudinal studies. Our RT-QuIC assay was highly reproducible, as illustrated by the two-center inter-laboratory comparison. In addition to the diagnostic value of this assay, the RT-QuIC skin assay may offer an opportunity to improve our understanding of the evolution of PD pathogenesis in the peripheral dermal tissue in living patients. We provide evidence for a moderate correlation of skin α-syn RT-QuIC seeding activity with disease duration, stage, and certain NMSs. Our promising results warrant further prospective exploration in larger patient populations including early or even prodromal stages of disease. To sum it up, skin biopsy RT-QuIC holds great promise as a reliable PD biomarker and may warrant broader implementation in clinical practice as well as usage as an outcome measure in disease-modifying clinical trials.

## Methods

### Study design

This prospective study was approved by the Ethics Committee of the University of Würzburg. All participants signed informed consent. Patients and controls were recruited at the Department of Neurology, University Hospital Würzburg. Patients with an expert clinical diagnosis of probable or established PD based on the criteria of the MDS were included^1^. The stage of disease was assessed using the H&Y scale^42^. NMSs were assessed using the German version of the NMSS for PD^43^. Patients were screened for MCI using the MoCA and for RBD using a single-question screening^44^. Controls were defined as probands without a history of parkinsonism or dementia and showing no signs of parkinsonism on neurological examination. Controls were represented by patients’ spouses or recruited from the patient populations treated in the neurology department for unrelated diseases (see Supplementary Table 1) and were assessed using the MDS research criteria for prodromal PD^45^ and the single-question screening for RBD^44^.

### Skin biopsy procedure

Skin punch biopsies (5 mm diameter) were performed at locations similar to those described in previous studies^5,19^, namely at C7 and Th10 paraspinally, lateral upper thigh and lower leg (the latter were only performed in a subset of subjects, see Supplementary Table 1). The biopsy specimens were divided into two halves perpendicularly to the epidermis to be processed by both laboratories separately, and both halves were flash-frozen in liquid nitrogen, blindly coded, and stored at −80 °C.

### RT-QuIC assay and lysate preparation

One half of each skin biopsy was analyzed at the Cleveland center. The RT-QuIC assay procedure, established based on our published protocol^19^, was modified and optimized for the small skin biopsy. The frozen skin biopsy specimen (~30 mg wet weight) was thawed and washed in ice-cold phosphate buffer at least three times until no visible blood remained. The skin tissue was cut into tiny pieces on a cold surface, followed by homogenization in 20 volumes of Dulbecco’s phosphate-buffered saline (PBS) supplemented with 1% Triton X-100, 150 mM NaCl, 5 mM EDTA, and proteinase inhibitors (Roche mini-cOmplete™, Roche Diagnostics, USA) with 1 mm zirconia beads agitated for 5 cycles of 1 min beating and 3 min cooling at 4 °C in a miniBeadbeater-16 device (BioSpec Products, USA). The resulting 5% skin lysate was further diluted 1:10 with PBS/N2 Gibco supplement (ThermoFisher, USA), from which 2 µl of the diluted lysate (equivalent to 200-fold dilution of skin tissue [w/v]) was added to the reaction mixture containing 40 mM NaPO4, pH 8, 170 mM NaCl, 20 µM thioflavin T (ThT), 10 µg commercial recombinant α-syn (rPeptide, USA), and 6 silica glass beads (OPS Diagnostics, USA) in a total volume of 100 µl per well of a black 96-well plate with a clear bottom (Nunc, ThermoFisher, USA). Four replicate reactions were made for each skin sample. The plate was sealed with Nunc clear sealing film and incubated at 42 °C in a BMG FLUOstar Omega plate reader (BMG Labtech, USA) with cycles of 1 min shaking (400 rpm, double orbital) and 1 min rest throughout the assay. ThT fluorescence (in arbitrary units [AU] at 450 ± 10 nm excitation and 480 ± 10 nm emission; bottom read) was recorded every 45 min for a total run of 50 h. Raw data were normalized to a percentage of the maximal fluorescence response (260,000 AU). A sample was considered positive when at least two out of four replicates crossed the threshold of background fluorescence plus 5 standard deviations (yielding a ~9.6% cut-off threshold) within 50 h. For each positive sample, the average ThT fluorescence intensity of the positive replicates was plotted against time. For each negative sample, the average fluorescence intensity of the negative replicates was plotted against time. For Spearman–Kärber analyses to obtain the seeding dose giving ThT positivity in 50% of replicate wells (SD_50_), threefold serial endpoint dilutions of a skin sample were prepared. Each diluted sample was used to seed RT-QuIC reactions in quadruplicate. ThT fluorescence from individual replicates for each dilution was plotted against time. SD_50_ per mg skin tissue was calculated based on the Spearman–Kärber equations as described^18^.

The other half of each biopsy was assessed at the Würzburg center using a similar assay procedure as described above with some modifications. Briefly, two 7-mm steel beads were used to homogenize skin tissue using the TissueLyser LT device (Qiagen, Germany) to prepare 5% skin lysates. The lysates were further diluted tenfold and 2 μl was added to a reaction mix composed of 0.1 M sodium phosphate buffer pH 7, 500 mM NaCl, 10 µM ThT, 20 µg recombinant α-syn synthesized in-house (see below), and 6 silica glass beads (OPS Diagnostics) in a total volume of 100 μl. Assay conditions were optimized using PD and healthy control brain lysates (Brain Bank Center Würzburg, BrainNet Europe Brain Bank Consortium Network). The 96-well plates were incubated at 37 °C with cycles of 1 min circular shaking 432 rpm and 14 min rest with fluorescence readings every 45 min in a Tecan Infinite M200 microplate reader (Tecan Group Ltd., Switzerland). Raw data were normalized to a percentage of the maximal fluorescence response (60,000 AU). The sample was considered to be positive if the fluorescence signal of at least 2 of the 4 replicates exceeded the 10% cut-off threshold. The assays were run for 60–80 h.

### Recombinant α-syn protein expression

An in-house synthesized recombinant α-syn protein was used in the Würzburg-based RT-QuIC assay. A bacterial plasmid carrying human α-syn TAT mutant gene (kindly provided by Professor Roucou, University of Sherbrooke, Canada) was used to avoid cysteine misincorporation at codon 136 during bacterial expression and subsequent α-syn dimerization^46,47^. The protein was overexpressed in BL21(DE3) *Escherichia coli* cells. Bacteria were grown at 37 °C to OD_600nm_ of 0.7 in LB media, before the expression was induced for 2 h upon addition of 0.44 mM isopropyl β-d-1-thiogalactopyranoside. The bacterial pellets were resuspended in lysis buffer containing 300 mM NaCl, 50 mM sodium dihydrogenphosphate (pH 7.4), 1 mM phenylmethylsulfonyl fluoride, 0.1 mM tris-(2-carboxyethyl) phosphine (TCEP), and 1 mg/ml lysozyme. Cells were lysed by sonication and the lysate was cleared by centrifugation for 30 min at 38,000 × *g*, 4 °C. The cleared lysate was mixed with Ni-NTA agarose (Qiagen) and incubated overnight at 4 °C. Ni-NTA beads were transferred into a gravity flow chromatography column, washed with 40 volumes of lysis buffer. The protein was eluted with 125 mM NaCl, 300 mM imidazole, 0.1 mM TCEP, and 25 mM sodium dihydrogenphosphate (pH 7.4). Roughly 15 mg recombinant a-syn were purified from 1 liter of expression culture. Fractions containing the target protein were pooled and further purified by size exclusion chromatography (HiLoad Superdex 75 16/600 pg, Cytiva, USA) in PBS (pH 7.4). Prior to long-term storage at −80 °C, the protein was diluted to 5 mg/ml and flash-frozen in liquid nitrogen.

### Parameters of RT-QuIC seeding activity and skin RT-QuIC score

In addition to the above described positive/negative outcome for each sample, the following parameters were calculated: mean ThT fluorescence at the end of 50-h (Cleveland) or 70-h (Würzburg) runs, referred to as final percentage of ThT fluorescence, the time (h) for a reaction to reach 50% of the maximum fluorescence (T50), and duration of lag phase (the reaction time [h] required to cross the fluorescence threshold). Assays with subthreshold signals were assigned T50 and lag phase equal to the duration of RT-QuIC runs (≥50 h).

To characterize the validity of α-syn seeding activity across multiple experiments and skin biopsy sites, a unified skin score of α-syn seeding activity was calculated. The rate of positive replicates out of four replicates per sample in both Cleveland and Würzburg assays was calculated for each biopsy site (i.e., 4/4 = 1, 3/4 = 0.75, 2/4 = 0.5, 1/4 = 0.25, and 0/4 = 0), as was similarly described in previous studies^17,48^. The rate of positive replicates was then averaged for each subject across all biopsy sites resulting in a laboratory-specific score for each participant, ranging from 0 to 1. The scores positively correlated with the mean final percentage of ThT fluorescence as a direct parameter of RT-QuIC seeding activity (Spearman’s *ρ* = 0.79, *p* < 0.001). The average of the laboratory-specific scores is further referred to as the “skin RT-QuIC score.” A score of at least 0.5 (similarly to conventionally used 2 out of 4 replicates) was considered clearly positive, and a score of ≤0.25 was considered negative. An intermediate score between 0.25 and 0.5 was included to account for a possible uneven distribution of α-syn seeding activity across biopsy sites.

### Statistical analysis

Statistical analyses were conducted using the R software (RStudio version 1.3.1073; Boston, MA, USA)^49^ and GraphPad Prism version 9.0.0 (San Diego, CA, USA). For intergroup comparisons of normally distributed data (such as percentage of ThT fluorescence), an unpaired two-sided *t* test was used and data were represented by means and standard deviations. Fisher’s exact test was used for categorical data. In the case of subgroup comparisons involving the skin RT-QuIC score, two-sided Mann–Whitney *U*-Test was applied, and data were illustrated by medians and interquartile ranges. To correlate skin RT-QuIC score with numerical clinical parameters, such as NMSS or MoCA, Spearman’s *ρ* correlation coefficient was applied. For inter-laboratory reproducibility estimations, Cohen’s kappa coefficients calculated using “kappa2” function of the “irr” library and Spearman’s *ρ* correlation coefficient using R were applied. Figures were created in ggplot2 version 3.3.2 and GraphPad.

### Reporting summary

Further information on research design is available in the Nature Research Reporting Summary linked to this article.

## Supplementary information

Supplementary Information

Reporting Summary

## Data Availability

The raw data that support the findings of this study are available from the corresponding authors upon reasonable request.
