# Supplementary material for: Diagnostic value of skin RT-QuIC in Parkinson’s disease: a two-laboratory study
Source: NPJ Parkinsons Dis. 2021 Nov 15;7:99. doi: 10.1038/s41531-021-00242-2 (PMC8593128; doi:10.1038/s41531-021-00242-2)
Supplement: Supplementary file 1 — Supplementary Information [file 41531_2021_242_MOESM1_ESM.pdf]

Supplementary Table 1: Detailed clinical data and RT-QuIC results of all participants.

ID	Diagnosis	Sex	H & Y stage	Age at diagnosis	Age at biopsy	PD duration (years)	Family history (any)	Fanily history (first-degree)	Concomitant disease	NMS Scale, total score	Number of key NMS reported	RBD	Depression	MoCA	Daytime sleepiness	RLS	Constipation	Urinary dysfunction	Hyposmia (subjective)	Insomnia	Erectile dysfunction	Orthostatic dysfunction	Motor subtype	MDS diagnostic criteria
PD1	PD	male	3	51	65	13,6	yes	yes	none	88	6	yes	no	27	no	no	yes	yes	yes	yes	yes	yes	tremor dominant	clinically established
PD2	PD	male	2	30	58	27,8	yes	no	none	51	6	no	no		no	yes	yes	yes	yes	yes	yes	no	tremor dominant	clinically established
PD3	PD	female	3	50	66	16,0	no	no	spinal disc herniation coxarthrosis, femur osteonecrosis	127	5	yes	no	28	yes	yes	no	no	yes	yes	yes	no	akinetic-rigid	clinically established
PD4	PD	male	2	49	60	10,7	no	no	depression	44	5	yes	yes	29	yes	no	no	no	yes	yes	no	no	akinetic-rigid	clinically established
PD5	PD	male	2	42	51	9,3	no	no	none	31	3	no	no	28	yes	yes	no	yes	no	no	no	no	akinetic-rigid	clinically established
PD6	PD	male	2	45	60	14,5	yes	no	none	64	5	yes	no	27	no	no	yes	yes	yes	yes	no	no	akinetic-rigid	clinically established
PD7	PD	male	1	50	56	6,2	no	no	none	27	0	no	no	27	no	no	no	no	no	no	no	no	tremor dominant	clinically established
PD8	PD	male	2	66	71	5,1	yes	yes	obstructive sleep apnoe, breast cancer	12	4	yes	no	28	no	no	yes	no	yes	yes	no	no	akinetic-rigid	clinically established
PD9	PD	male	2	57	62	5,4	yes	no	hypothyreosis, arterial hypertension	27	5	no	no	28	yes	no	no	yes	yes	yes	no	yes	tremor dominant	clinically established
PD10	PD	male	2	49	66	16,8	yes	yes	polyneuropathy	14	4	yes	no	20	no	no	yes	no	yes	no	no	no	akinetic-rigid	clinically established
PD11	PD	female	5	50	75	24,7	no	no	none	108	9	yes	no	25	yes	yes	yes	yes	yes	yes	no	yes	akinetic-rigid	clinically established
PD12	PD	female	3	71	77	6,5	no	no	lumbar spinal ste osis, iron and B12 deficiency	67	6	yes	no	24	no	yes	yes	yes	yes	no	no	no	akinetic-rigid	clinically established
PD13	PD	male	2	63	74	10,8	no	no	none	62	6	yes	no	27	yes	yes	yes	yes	yes	no	no	no	akinetic-rigid	clinically established
PD14	PD	male	2	77	78	0,7	no	no	prostate hyperplasia, PNP	46	2	no	no	27	no	yes	no	yes	no	no	no	no	akinetic-rigid	clinically probable
PD15	PD	male	2	60	65	5,5	no	no	carpal tunnel syndrome	34	1	yes	no	26	no	no	no	no	no	no	no	no	akinetic-rigid	clinically established
PD16	PD	male	4	73	80	7,4	no	no	cervical spinal stenosis	24	2		no	25	no	no	no	yes	no	no	no	no	akinetic-rigid	clinically probable
PD17	PD	male	3	59	70	11,1	no	no	essential tremor	63	6	yes	no	28	no	yes	no	yes	yes	yes	yes	yes	tremor dominant	clinically established
PD18	PD	female	3	55	73	18,1	no	no	none	121	8	yes	no	27	yes	yes	yes	yes	yes	yes	no	yes	tremor dominant	clinically established
PD19	PD	male	2	74	74	0,2	no	no	rheumathoid arthritis, arterial hypertension, spinal stenosis	27	2	no	no		no	no	no	no	yes	no	no	no	akinetic-rigid	clinically probable
PD20	PD	female	2	55	67	11,9	no	no	transitory ischemic attack, arterial hypertension, diabetes mellitus type 2, bronchial sthma, sleep apnoe, myocarditis, spinal disc prolapse	25	1	no	no	28	yes	no	no	no	no	no	no	no	mixed	clinically established
PD21	PD	male	3	81	81	0,2	yes	yes	arterial hypertension, aortic dissection, hypothyroidism	16	4	no	no		no	no	yes	no	yes	yes	no	no	akinetic-rigid	clinically probable
PD22	PD	male	3	36	55	18,6	no	no	meningioma, carpal tunnel syndrome	45	5	yes	no	25	no	no	yes	no	yes	yes	no	no	akinetic-rigid	clinically established
PD23	PD	male	4	51	74	23,0	no	no	arterial hypertension, depression, spinal disc prolapse	125	7	yes	yes	25	yes	yes	yes	no	yes	no	no	no	akinetic-rigid	clinically established
PD24	PD	male	2	53	60	7,2	yes	yes	sleep apnoe, arterial hypertension	24	1	no	no	28	no	no	no	no	no	yes	no	no	akinetic-rigid	clinically probable
PD25	PD	female	4	47	66	19,0	yes	no	chronic obstructive pulmonary disease, arterial hypertension	10	1	no	no	27	no	no	no	no	yes	no	no	no	tremor dominant	clinically established
PD26	PD	male	2	37	51	14,3	yes	yes	depression	21	5	yes	yes	28	yes	no	no	yes	yes	no	yes	no	mixed	clinically established
PD27	PD	female	3	64	72	8,5	yes	yes	rectal carcinoma, benign skin tumor	36	5	yes	no	26	no	no	no	yes	yes	yes	no	yes	tremor dominant	clinically established
PD28	PD	male	2	41	53	12,1	no	no	diabetes mellitus type 2, depression	35	4	yes	yes	27	no	no	no	no	yes	no	no	yes	akinetic-rigid	clinically established
PD29	PD	female	2	58	73	14,6	yes	yes	macular degeneration	51	4	no	no	29	yes	yes	yes	no	yes	no	no	no	mixed	clinically established
PD30	PD	male	2	51	58	7,3	no	no	diabetes mellitus type 2, arterial hypertonie, bronchial asthma, sleep apnea, adipositas permagna	7	4	yes	no		yes	no	no	no	no	yes	no	no	mixed	clinically established
PD31	PD	female	3	49	65	16,1	no	no	diabetes mellitus type 2, arterial hypertonie	15	4	no	no	24	yes	no	no	no	yes	yes	no	no	akinetic-rigid	clinically established
PD32	PD	male	2	70	74	3,7	no	no	rectal carcinoma	39	5	no	no	27	yes	yes	no	yes	no	yes	no	yes	akinetic-rigid	clinically established
PD33	PD	male	3	68	79	10,7	no	no	none	44	5	yes	no	26	yes	no	yes	yes	no	yes	no	no	mixed	clinically established
PD34	PD	female	2	53	73	19,5	no	no	depression	43	6	yes	yes	24	yes	no	no	yes	no	no	no	yes	akinetic-rigid	clinically established

ID	Diagnosis	Final result Cleveland	Skin RT-QuIC score Cleveland	Final result Würzburg	Skin RT-QuIC score Würzburg	Final result (both sites)	Skin RT-QuIC score (both sites)	C7 RT-QuIC score (both sites)	Th10 RT-QuIC score (both sites)	Thigh RT-QuIC score (both sites)	Lower leg RT-QuIC score (both sites)	C7 RT-QuIC result (Cleveland)	Th10 RT-QuIC result (Cleveland)	Thigh RT-QuIC result (Cleveland)	Lower leg RT-QuIC result (Cleveland)	C7 RT-QuIC score (Cleveland)	Th10 RT-QuIC score (Cleveland)	Thigh RT-QuIC score (Cleveland)	Lower leg RT-QuIC score (Cleveland)	Mean T50, hours (Cleveland	Mean lag phase duration, hours (Cleveland)	C7 RT-QuIC result (Würzburg)	Th10 RT-QuIC result (Würzburg)	Thigh RT-QuIC result (Würzburg)	Lower leg RT-QuIC result (Würzburg)	C7 RT-QuIC score (Würzburg)	Th10 RT-QuIC score (Würzburg)	Thigh RT-QuIC score (Würzburg)	Lower leg RT-QuIC score (Würzburg)
PD1	PD	pos	1,00	pos	1,00	pos	1,00	1,00	1,00	1,00	1,00	pos	pos	pos	pos	1,00	1,00	1,00	1,00	17,2	9,4	pos	pos	pos	pos	1,00	1,00	1,00	1,00
PD2	PD	pos	1,00	pos	0,88	pos	0,94	1,00	1,00	0,88	0,88	pos	pos	pos	pos	1,00	1,00	1,00	1,00	22,1	11,5	pos	pos	pos	pos	1,00	1,00	0,75	0,75
PD3	PD	pos	0,75	pos	0,69	pos	0,72	0,00	1,00	1,00	0,88	neg	pos	pos	pos	0,00	1,00	1,00	1,00	34,0	27,6	neg	pos	pos	pos	0,00	1,00	1,00	0,75
PD4	PD	pos	0,53	pos	0,50	pos	0,51	0,13	0,43	0,63	0,88	neg	pos	neg	pos	0,25	0,60	0,25	1,00	39,0	36,9	neg	neg	pos	pos	0,00	0,25	1,00	0,75
PD5	PD	interm	0,33	interm	0,31	interm	0,32	0,00	0,00	1,00	0,29	neg	neg	pos	interm	0,00	0,00	1,00	0,33	46,6	44,0	neg	neg	pos	neg	0,00	0,00	1,00	0,25
PD6	PD	pos	0,91	pos	0,81	pos	0,86	1,00	0,81	0,63	1,00	pos	pos	pos	pos	1,00	0,88	0,75	1,00	19,8	12,6	pos	pos	pos	pos	1,00	0,75	0,50	1,00
PD7	PD	neg	0,13	neg	0,06	neg	0,09	0,00	0,13	0,13	0,13	neg	neg	neg	neg	0,00	0,00	0,25	0,25	50,0	50,0	neg	neg	neg	neg	0,00	0,25	0,00	0,00
PD8	PD	pos	1,00	pos	0,88	pos	0,94	0,88	1,00	1,00	0,88	pos	pos	pos	pos	1,00	1,00	1,00	1,00	22,7	17,1	pos	pos	pos	pos	0,75	1,00	1,00	0,75
PD9	PD	pos	0,63	pos	0,63	pos	0,63	0,88	0,00	0,75	0,88	pos	neg	pos	pos	0,75	0,00	0,75	1,00	37,9	30,1	pos	neg	pos	pos	1,00	0,00	0,75	0,75
PD10	PD	pos	1,00	pos	0,88	pos	0,94	1,00	1,00	0,75	1,00	pos	pos	pos	pos	1,00	1,00	1,00	1,00	28,9	21,5	pos	pos	pos	pos	1,00	1,00	0,50	1,00
PD11	PD	pos	1,00	pos	1,00	pos	1,00	1,00	1,00	1,00	1,00	pos	pos	pos	pos	1,00	1,00	1,00	1,00	28,5	20,4	pos	pos	pos	pos	1,00	1,00	1,00	1,00
PD12	PD	pos	1,00	pos	0,92	pos	0,96	1,00	0,88	1,00		pos	pos	pos		1,00	1,00	1,00		28,2	19,6	pos	pos	pos		1,00	0,75	1,00	
PD13	PD	pos	1,00	pos	0,94	pos	0,97	1,00	0,88	1,00	1,00	pos	pos	pos	pos	1,00	1,00	1,00	1,00	24,9	17,2	pos	pos	pos	pos	1,00	0,75	1,00	1,00
PD14	PD	neg	0,08	neg	0,08	neg	0,08	0,13	0,13	0,00		neg	neg	neg		0,00	0,25	0,00		49,5	49,5	neg	neg	neg		0,25	0,00	0,00	
PD15	PD	pos	1,00	pos	1,00	pos	1,00	1,00	1,00	1,00	1,00	pos	pos	pos	pos	1,00	1,00	1,00	1,00	20,1	11,5	pos	pos	pos	pos	1,00	1,00	1,00	1,00
PD16	PD	pos	1,00	pos	1,00	pos	1,00	1,00	1,00	1,00		pos	pos	pos		1,00	1,00	1,00		19,4	11,1	pos	pos	pos		1,00	1,00	1,00	
PD17	PD	interm	0,48	pos	0,75	pos	0,61	1,00	0,25	0,41	0,79	pos	neg	neg	pos	1,00	0,00	0,08	0,83	36,3	30,8	pos	pos	pos	pos	1,00	0,50	0,75	0,75
PD18	PD	pos	1,00	pos	0,67	pos	0,83	1,00	0,75	0,75		pos	pos	pos		1,00	1,00	1,00		23,3	13,2	pos	pos	pos		1,00	0,50	0,50	
PD19	PD	pos	0,75	pos	0,50	pos	0,63	0,63	0,13	0,75	1,00	pos	neg	pos	pos	0,75	0,25	1,00	1,00	36,9	31,6	pos	neg	pos	pos	0,50	0,00	0,50	1,00
PD20	PD	neg	0,25	interm	0,44	interm	0,34	0,13	0,13	0,25	0,88	neg	neg	neg	pos	0,00	0,00	0,25	0,75	46,3	42,8	neg	neg	neg	pos	0,25	0,25	0,25	1,00
PD21	PD	pos	0,67	pos	0,75	pos	0,71	0,81	1,00	0,31		pos	pos	neg		0,75	1,00	0,25		42,9	38,7	pos	pos	interm		0,88	1,00	0,38	
PD22	PD	pos	0,88	pos	0,96	pos	0,92	1,00	0,88	0,88		pos	pos	pos		1,00	0,88	0,75		28,0	21,2	pos	pos	pos		1,00	0,88	1,00	
PD23	PD	pos	1,00	pos	1,00	pos	1,00	1,00	1,00	1,00		pos	pos	pos		1,00	1,00	1,00		22,4	15,8	pos	pos	pos		1,00	1,00	1,00	
PD24	PD	pos	0,71	pos	0,88	pos	0,79	0,81	0,56	1,00		pos	interm	pos		0,75	0,38	1,00		27,9	20,7	pos	pos	pos		0,88	0,75	1,00	
PD25	PD	pos	0,67	pos	0,83	pos	0,75	1,00	1,00	0,25		pos	pos	neg		1,00	1,00	0,00		30,0	25,5	pos	pos	pos		1,00	1,00	0,50	
PD26	PD	interm	0,44	interm	0,42	interm	0,43	0,13	0,19	0,98		neg	neg	pos		0,25	0,13	0,95		53,2	51,3	neg	neg	pos		0,00	0,25	1,00	
PD27	PD	pos	0,88	pos	0,75	pos	0,81	0,56	1,00	0,88		pos	pos	pos		0,63	1,00	1,00		29,4	22,1	pos	pos	pos		0,50	1,00	0,75	
PD28	PD	pos	0,67	pos	0,67	pos	0,67	1,00	1,00			pos	pos			1,00	1,00			21,3	13,0	pos	pos			1,00	1,00		
PD29	PD	pos	0,79	pos	0,96	pos	0,88	0,63	1,00	1,00		interm	pos	pos		0,38	1,00	1,00		26,9	19,5	pos	pos	pos		0,88	1,00	1,00	
PD30	PD	pos	1,00	pos	0,92	pos	0,96	0,88	1,00	1,00		pos	pos	pos		1,00	1,00	1,00		25,8	21,3	pos	pos	pos		0,75	1,00	1,00	
PD31	PD	pos	0,59	pos	0,81	pos	0,70	1,00	0,81	0,44	0,56	pos	pos	neg	interm	1,00	0,88	0,13	0,38	42,6	36,9	pos	pos	pos	pos	1,00	0,75	0,75	0,75
PD32	PD	neg	0,17	neg	0,17	neg	0,17	0,19	0,19	0,13		neg	neg	neg		0,13	0,13	0,25		54,6	54,1	neg	neg	neg		0,25	0,25	0,00	
PD33	PD	pos	1,00	pos	0,83	pos	0,92	0,88	0,88	1,00		pos	pos	pos		1,00	1,00	1,00		32,4	24,6	pos	pos	pos		0,75	0,75	1,00	
PD34	PD	pos	1,00	pos	1,00	pos	1,00	1,00		1,00		pos		pos		1,00		1,00		33,0	26,6	pos		pos		1,00	0,00	1,00	

ID	Diagnosis	Sex	H & Y stage	Age at diagnosis	Age at biopsy	PD duration (years)	Family history (any)	Fanily history (first-degree)	Concomitant disease	NMS Scale, total score	Number of key NMS reported	RBD	Depression	MoCA	Daytime sleepiness	RLS	Constipation	Urinary dysfunction	Hyposmia (subjective)	Insomnia	Erectile dysfunction	Orthostatic dysfunction	Motor subtype	MDS diagnostic criteria
CTL1	control	male			55			no	chronic inflammatory demyelinating polyneuropathy, MGUS, osteoporosis, arterial hypertension, urge incontinence															
CTL2	control	male			79			no	myasthenia gravis			no	no		no		no	yes	yes, subjectively		no	no		
CTL3	control	male			61			no	inflammatory polyneuropathy of uncertain type			no	no		yes		no	no	yes, subjectively		no	yes, subjectively		
CTL4	control	male			51			no	stroke, spinal muscular atrophyll fiber neuropathy, aortic aneurism			no	no		no		no	no	no		yes	no		
CTL5	control	male			66			no	polyneuropathy, ATTR-amyloidosis															
CTL6	control	female			78			no	epilepsy, struma multinpdosa, arterial hypertension			no	no		no		no	no	no			no		
CTL7	control	female			69			no	spinal muscular atrophyll fiber neuropathy			no	no		yes		no	no	no		no	yes, subjectively		
CTL8	control	male			57			no	spinal muscular atrophyll fiber neuropathy															
CTL9	control	female			65			no	spinal muscular atrophyll fiber neuropathy			no	yes		yes		no	yes	no			no		
CTL10	control	male			62			no	polyneuropathy, spinal stenosis			no	no		no		no	no	no		no	no		
CTL11	control	female			70			no	diabetes mellitus type 2, breast cancer			no	no		no		no	no	no		no	no		
CTL12	control	female			62			no	spinal muscular atrophyll fiber neuropathy, spinal stenosis, depression			no	yes		no		no	no	no		no	no		
CTL13	control	female			55			no	mysthenia, multiple sclerosis, arterial hypertension, Hashimoto thyreoiditis			yes	yes		yes		no	no	no			yes, subjectively		
CTL14	control	male			59			no	stiff person syndrome			no	no		no		no	no	no		no	no		
CTL15	control	female			61			no	multiple sclerosis, depression, anxiety			no	yes		no		no	no	no			no		
CTL16	control	male			77			no	myasthenia gravis			no	no		no		no	no	yes, subjectively		no	no		
CTL17	control	male			59			no	spinal muscular atrophy			no	no		no		no	no	no		no	no		
CTL18	control	male			58			no	autoimmune myelitis			no	no		no		no	no	yes, subjectively		no	no		
CTL19	control	female			61			no	herpes zoster meningitis			no	no		no		no	no	yes, subjectively		no	no		
CTL20	control	female			65			no	spinal muscular atrophy			no	no		no		no	yes	no			no		
CTL21	control	female			55			no	multiple sclerosis			no	yes		yes		no	no	no		no	no		
CTL22	control	male			71			no	vasculitic polyneuropathy			no	no		no		no	no	no		no	no		
CTL23	control	female			50			no	multiple sclerosis, arterial hypertension			no	no		no		no	yes	yes, subjectively			no		
CTL24	control	male			57			no	transitory ischemic attack, aortic stenosis			no	no		no		no	no	no		no	no		
CTL25	control	male			66			no	diffuse large B-cell lymphoma			no	no		no		no	no	no		no	no		
CTL26	control	female			61			no	myasthenia gravis			no	no		no		no	no	no		no	no		
CTL27	control	female			79			no	orthostatic tremor			no	no		no		no	yes	no		no	no		
CTL28	control	male			60			no	stroke, arterial hypertension			no	no		no		no	no	no		no	no		
CTL29	control	male			63			no	Morvan's syndrome, diabetes mellitus type 2, arterial hypertension			no	no		no		no	no	no		no	no		
CTL30	control	male			71			no	none			yes	no		no		no	no	yes, objectified by Sniffin Sticks		no	no		

ID	Diagnosis	Final result Cleveland	Skin RT-QuIC score Cleveland	Final result Würzburg	Skin RT-QuIC score Würzburg	Final result (both sites)	Skin RT-QuIC score (both sites)	C7 RT-QuIC score (both sites)	Th10 RT-QuIC score (both sites)	Thigh RT-QuIC score (both sites)	Lower leg RT-QuIC score (both sites)	C7 RT-QuIC result (Cleveland)	Th10 RT-QuIC result (Cleveland)	Thigh RT-QuIC result (Cleveland)	Lower leg RT-QuIC result (Cleveland)	C7 RT-QuIC score (Cleveland)	Th10 RT-QuIC score (Cleveland)	Thigh RT-QuIC score (Cleveland)	Lower leg RT-QuIC score (Cleveland)	Mean T50, hours (Cleveland	Mean lag phase duration, hours (Cleveland)	C7 RT-QuIC result (Würzburg)	Th10 RT-QuIC result (Würzburg)	Thigh RT-QuIC result (Würzburg)	Lower leg RT-QuIC result (Würzburg)	C7 RT-QuIC score (Würzburg)	Th10 RT-QuIC score (Würzburg)	Thigh RT-QuIC score (Würzburg)	Lower leg RT-QuIC score (Würzburg)
CTL1	control	neg	0,13	neg	0,00	neg	0,06	0,13	0,00	0,00		neg	neg			0,25	0,00	0,00				neg	neg			0,00	0,00		
CTL2	control	neg	0,09	neg	0,19	neg	0,14	0,00	0,13	0,31	0,13	neg	neg	neg	neg	0,00	0,00	0,38	0,00			neg	neg	neg	neg	0,00	0,25	0,25	0,25
CTL3	control	neg	0,00	neg	0,08	neg	0,04	0,00	0,00	0,13		neg	neg	neg		0,00	0,00	0,00				neg	neg	neg		0,00	0,00	0,25	
CTL4	control	neg	0,00	neg	0,13	neg	0,06	0,00	0,13			neg	neg			0,00	0,00					neg	neg			0,00	0,25		
CTL5	control	neg	0,08	neg	0,13	neg	0,10	0,19	0,13	0,00		neg	neg	neg		0,25	0,00	0,00				neg	neg	neg		0,13	0,25	0,00	
CTL6	control	neg	0,25	neg	0,13	neg	0,19	0,13	0,19	0,25		neg	neg	interm		0,13	0,25	0,38				neg	neg	neg		0,13	0,13	0,13	
CTL7	control	neg	0,00	neg	0,25	neg	0,13	0,13	0,00			neg				0,00						neg				0,25			
CTL8	control	neg	0,00	neg	0,00	neg	0,00	0,00				neg				0,00						neg				0,00			
CTL9	control	pos	0,68	pos	0,60	pos	0,64	0,64				pos				0,68						pos				0,60			
CTL10	control	neg	0,00	neg	0,17	neg	0,08	0,00	0,13	0,13		neg	neg	neg		0,00	0,00	0,00				neg	neg	neg		0,00	0,25	0,25	
CTL11	control	neg	0,15	interm	0,29	neg	0,22	0,00	0,25	0,41		neg	neg	interm		0,00	0,00	0,45				neg	pos	interm		0,00	0,50	0,38	
CTL12	control	neg	0,00	neg	0,25	neg	0,13	0,13	0,00			neg				0,00						neg				0,25			
CTL13	control	pos	0,62	pos	0,50	pos	0,56	0,56	0,46	0,65		pos	pos	pos		0,50	0,68	0,68				pos	neg	pos		0,63	0,25	0,63	
CTL14	control	neg	0,25	neg	0,17	neg	0,21	0,25	0,13	0,25		neg	neg	neg		0,25	0,25	0,25				neg	neg	neg		0,25	0,00	0,25	
CTL15	control	neg	0,25	neg	0,13	neg	0,19	0,06	0,13	0,38		neg	neg	pos		0,00	0,25	0,50				neg	neg	neg		0,13	0,00	0,25	
CTL16	control	neg	0,25	neg	0,25	neg	0,25	0,19	0,38	0,19		neg	interm	neg		0,25	0,38	0,13				neg	interm	neg		0,13	0,38	0,25	
CTL17	control	neg	0,17	neg	0,08	neg	0,13	0,13	0,13	0,13		neg	neg	neg		0,25	0,25	0,00				neg	neg	neg		0,00	0,00	0,25	
CTL18	control	neg	0,21	neg	0,25	neg	0,23	0,06	0,13	0,50		neg	neg	pos		0,13	0,00	0,50				neg	neg	pos		0,00	0,25	0,50	
CTL19	control	neg	0,25	neg	0,08	neg	0,17	0,13	0,25	0,13		neg	neg	neg		0,25	0,25	0,25				neg	neg	neg		0,00	0,25	0,00	
CTL20	control	neg	0,17	neg	0,17	neg	0,17	0,13	0,13	0,25		neg	neg	neg		0,25	0,00	0,25				neg	neg	neg		0,00	0,25	0,25	
CTL21	control	interm	0,35	neg	0,21	interm	0,28	0,06	0,40	0,38		neg	interm	pos		0,13	0,43	0,50				neg	interm	neg		0,00	0,38	0,25	
CTL22	control	neg	0,00	neg	0,00	neg	0,00	0,00	0,00	0,00		neg	neg	neg		0,00	0,00	0,00				neg	neg	neg		0,00	0,00	0,00	
CTL23	control	neg	0,25	neg	0,21	neg	0,23	0,00	0,25	0,44		neg	neg	pos		0,00	0,13	0,63				neg	interm	neg		0,00	0,38	0,25	
CTL24	control	neg	0,00	neg	0,25	neg	0,13	0,13	0,13	0,13		neg	neg	neg		0,00	0,00	0,00				neg	neg	neg		0,25	0,25	0,25	
CTL25	control	neg	0,00	neg	0,25	neg	0,13	0,13	0,13	0,13		neg	neg	neg		0,00	0,00	0,00				neg	neg	neg		0,25	0,25	0,25	
CTL26	control	neg	0,17	neg	0,17	neg	0,17	0,13	0,13	0,25		neg	neg	neg		0,25	0,00	0,25				neg	neg	neg		0,00	0,25	0,25	
CTL27	control	neg	0,21	neg	0,25	neg	0,23	0,13	0,44	0,13		neg	interm	neg		0,00	0,38	0,25				neg	pos	neg		0,25	0,50	0,00	
CTL28	control	neg	0,00	neg	0,00	neg	0,00	0,00	0,00	0,00		neg	neg	neg		0,00	0,00	0,00				neg	neg	neg		0,00	0,00	0,00	
CTL29	control	neg	0,00	neg	0,08	neg	0,04	0,13	0,00	0,00		neg	neg	neg		0,00	0,00	0,00				neg	neg	neg		0,25	0,00	0,00	
CTL30	control	interm	0,42	pos	0,67	pos	0,54	0,00	0,75	0,88		neg	pos	pos		0,00	0,50	0,75				neg	pos	pos		0,00	1,00	1,00	

PD1-PD34 = study participants with Parkinson's disease #1-34. CTL1-CTL30 = control subjects #1-30. The headings are color-coded: clinical data is orange. NMS = non-motor symptoms, RLS = restless legs syndrome, RBD = REM-Sleep behavior disorder, MoCA = Montreal cognitive assessment, MDS = movement disorders society. Headings for Cleveland assay results are coded violet, Wuerzburg assay results - green, pooled results - grey. RT-QuIC results are reported as RT-QuIC score ranging from 0 to 1. Additionally interpretation of the score is reported (neg = negative, interm = intermediate, pos = positive).
