# Supplementary material for: Diagnostic value of skin RT-QuIC in Parkinson’s disease: a two-laboratory study
Source: NPJ Parkinsons Dis. 2021 Nov 15;7:99. doi: 10.1038/s41531-021-00242-2 (PMC8593128; doi:10.1038/s41531-021-00242-2)
Supplement: Supplementary file 2 — Reporting Summary [file 41531_2021_242_MOESM2_ESM.pdf]

nature research

Corresponding author(s): Kathrin Doppler and Shu G. Chen

Last updated by author(s): Apr 15, 2021

Reporting Summary

Nature Research wishes to improve the reproducibility of the work that we publish. This form provides structure for consistency and transparency in reporting. For further information on Nature Research policies, see our Editorial Policies and the Editorial Policy Checklist.

Statistics

For all statistical analyses, confirm that the following items are present in the figure legend, table legend, main text, or Methods section.

n/a	Confirmed	
☐	☒	The exact sample size (*n*) for each experimental group/condition, given as a discrete number and unit of measurement
☐	☒	A statement on whether measurements were taken from distinct samples or whether the same sample was measured repeatedly
☐	☒	The statistical test(s) used AND whether they are one- or two-sided<br>*Only common tests should be described solely by name; describe more complex techniques in the Methods section.*
☒	☐	A description of all covariates tested
☒	☐	A description of any assumptions or corrections, such as tests of normality and adjustment for multiple comparisons
☐	☒	A full description of the statistical parameters including central tendency (e.g. means) or other basic estimates (e.g. regression coefficient) AND variation (e.g. standard deviation) or associated estimates of uncertainty (e.g. confidence intervals)
☐	☒	For null hypothesis testing, the test statistic (e.g. *F*, *t*, *r*) with confidence intervals, effect sizes, degrees of freedom and *P* value noted<br>*Give P values as exact values whenever suitable.*
☒	☐	For Bayesian analysis, information on the choice of priors and Markov chain Monte Carlo settings
☒	☐	For hierarchical and complex designs, identification of the appropriate level for tests and full reporting of outcomes
☐	☒	Estimates of effect sizes (e.g. Cohen's *d*, Pearson's *r*), indicating how they were calculated

Our web collection on statistics for biologists contains articles on many of the points above.

Software and code

Policy information about availability of computer code

Data collection	No specific code was used to collect data.
Data analysis	RStudio version 1.3.1073 (Boston, MA, USA) and GraphPad Prism version 9.0.0 (San Diego, CA, USA).

For manuscripts utilizing custom algorithms or software that are central to the research but not yet described in published literature, software must be made available to editors and reviewers. We strongly encourage code deposition in a community repository (e.g. GitHub). See the Nature Research guidelines for submitting code & software for further information.

Data

Policy information about availability of data

All manuscripts must include a data availability statement. This statement should provide the following information, where applicable:
- Accession codes, unique identifiers, or web links for publicly available datasets
- A list of figures that have associated raw data
- A description of any restrictions on data availability

The raw data that support the findings of this study are available from the corresponding authors upon reasonable request.

Field-specific reporting

Please select the one below that is the best fit for your research. If you are not sure, read the appropriate sections before making your selection.

☒ Life sciences ☐ Behavioural & social sciences ☐ Ecological, evolutionary & environmental sciences

For a reference copy of the document with all sections, see nature.com/documents/nr-reporting-summary-flat.pdf

Life sciences study design

All studies must disclose on these points even when the disclosure is negative.

Sample size	We assumed a conservative effect size of 0.8 based on results of previous studies of CSF alpha-synuclein RT-QuIC of other groups and our own immunohistochemical studies. Using a power of 0.9 and significance level of 0.05 we calculated using G.Power a needed number of participants of 34 for each group.
Data exclusions	Pre-established exclusion criteria - patients not reaching a clinical diagnostic certainty of at least probable PD (as per MDS criteria). We excluded 3 patients, whose diagnosis was reevaluated since recruitment (2 to MSA, 1 to vascular parkinsonism)
Replication	The samples were tested independently in 2 laboratories using different assay conditions.
Randomization	not relevant
Blinding	At the Wuerzburg site samples were blindly coded upon biopsy. All colleagues at the Cleveland site were blinded to the clinical data until finalization of results. Personnel performing the RT-QuIC assay at Wuerzburg site were blinded to clinical data.

Reporting for specific materials, systems and methods

We require information from authors about some types of materials, experimental systems and methods used in many studies. Here, indicate whether each material, system or method listed is relevant to your study. If you are not sure if a list item applies to your research, read the appropriate section before selecting a response.

Materials & experimental systems

n/a	Involved in the study	
☒	☐	Antibodies
☒	☐	Eukaryotic cell lines
☒	☐	Palaeontology and archaeology
☒	☐	Animals and other organisms
☐	☒	Human research participants
☒	☐	Clinical data
☒	☐	Dual use research of concern

Methods

n/a	Involved in the study	
☒	☐	ChIP-seq
☒	☐	Flow cytometry
☒	☐	MRI-based neuroimaging

Human research participants

Policy information about studies involving human research participants

Population characteristics	The PD cohort was not significantly older than controls. Gender distribution was in favor of males, in accordance with the population prevalence. Diagnosis was based on clinical criteria (only rarely supported by ancillary tests in selected probands - such as Sniffin Sticks - all such cases are mentioned in manuscript)
Recruitment	Participants were recruited from the in- and outpatient departments of the University Hospital of Wuerzburg. It is a specialized center for Parkinson's disease performing substantial number of DBS surgeries. Many of the patients had DBS surgery meaning a younger population with good-levodopa response but also making a genetic PD more likely (see Discussion)
Ethics oversight	Ethical review board of the University of Wuerzburg

Note that full information on the approval of the study protocol must also be provided in the manuscript.

April 2020
